# Case 4/2016: 32-Year-Old Female, with Critical Pulmonary Valve Stenosis. Operated at 4 Months of Age, in Normal Healing Evolution

**DOI:** 10.5935/abc.20160090

**Published:** 2016-06

**Authors:** Edmar Atik

**Affiliations:** Edmar Atik's Private Clinic, SP- Brazil

**Clinical Data:** Good clinical evolution was observed after correction of marked critical pulmonary valve stenosis, with central orifice with one (01) mm of diameter of valve opening, in a case of right heart failure and cyanosis by right to left shunt through foramen ovale, on an emergency basis, with circulatory arrest without cardiopulmonary bypass, at 4 months of age. On that occasion, a commissurotomy of the trivalvular pulmonary valve through the pulmonary trunk was performed. Mild systolic murmur in the pulmonary area, though less intense than before surgery, which remained audible until adolescence. Currently, the patient is able to carry out routine activities and does not refer any symptoms. The patient has led a normal life, having graduated in law at a traditional university with normal and well tolerated physical performance.

**Physical Examination:** good general state, eupneic, acyanotic, normal pulse. Weight: 57 Kgs. Height: 158 cm, right upper limb blood pressure: 110/70 mm Hg, HR: 74 bpm. The aorta was not palpated at the suprasternal notch. In the precordium, apex beat was not palpated and there were no systolic impulses. Heart sounds were normal and there was no audible cardiac murmur. The liver was not palpated and lungs were clear.

## Supplementary Exams

**Figure 1:**
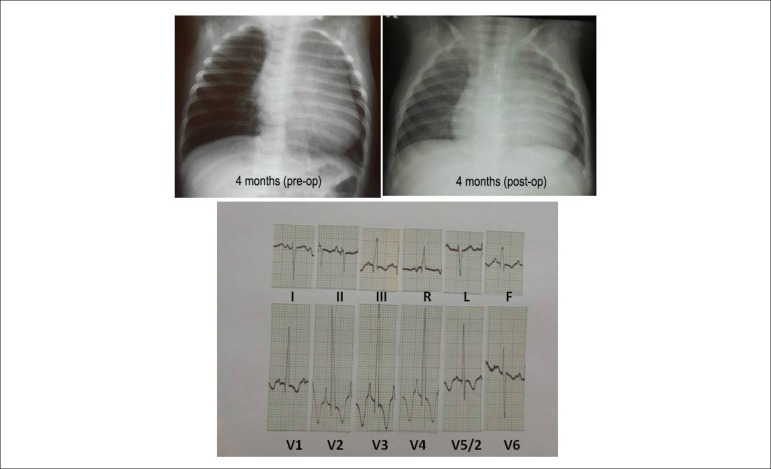
**Chest X-Rays** show enlarged cardiac area, with decreased pulmonary vasculature in periods pre and immediate to surgery for correction of pulmonary valve stenosis, and with electrocardiogram with marked overload of right cardiac cavities with signs of suprasystemic systolic pressure.

**Figure 2:**
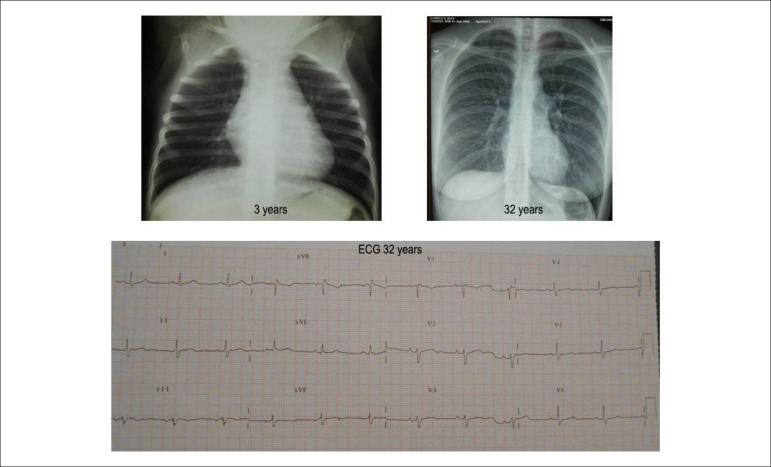
Chest X-Rays show slightly enlarged and fully normal cardiac area, respectively 3 and 32 years after surgical correction of pulmonary valve stenosis. Electrocardiogram with minimal end disorder of conduction through the right branch.

**Electrochardiogram** showed junctional rhythm and discreet end disorder of conduction through the right branch with complex rSr in V1 with normal duration of QRS of 0.094 seconds. AP = -30º; AQRS = -10º; AT = +10º (Figure 2). ECG, done before the surgery at 4 months old, highlighted the significant right ventricular and atrial overload with electrical signals of systolic pressure of the right ventricle greater than systemic pressure with depression of ST segment in the right precordials and QR waves from V1 to V5, largely negative T waves from V1 to V6 and peaked P wave with 5 to 6 mm amplitude from V2 to V4; AP = +50°, AQRS = + 170°, AT = -60°. (Figure 1).

**Chest X-Ray** showed cardiac area slightly enlarged, three years after surgery, and completely normal at 32 years of age, with normal pulmonary vasculature (Figure 2), in contrast with clear cardiomegaly from enlargement of right cavities in the periods of immediate pre and post-surgery (Figure 1).

**Echocardiogram** showed cardiac cavities with normal dimensions, normal biventricular function without valvular abnormalities, except for a slightly thickened pulmonary valve showing pressure gradient of 10 mm Hg and without any valve insufficiency. There was patent foramen ovale with minimal passage to the right atrium.

**Clinical Diagnosis:** Critical pulmonary valve stenosis operated with circulatory arrest in infant period, at 4 months of age, in evolution for long term anatomic and functional cure.

**Clinical Rationale:** Evolutionary clinical findings were consistent with the diagnosis of prior pulmonary valve stenosis given the persistency of end disorder of conduction through the right branch on electrocardiogram, electric expression of marked right ventricular overload prior to surgical correction. The absence of residual systolic murmur highlights good progress, and when this condition includes residual pressure gradient at the pulmonary valve, it should be under 10 mm Hg, as shown in the echocardiogram. Another favorable element is the normal size of the cardiac area in the chest X-Ray, which highlights anatomic and functional normality.

**Differential Diagnosis:** Operated congenital heart diseases which show the same clinical and laboratory aspects are those represented by interventricular and interatrial communication, patent ductus arteriosus, coarctation of the aorta, transposition of the great arteries, and anomalous pulmonary venous drainage, among the main ones.

**Conduct:** In view of the anatomic and functional normalization, a healthy and normal life with assurance of the capability to perform any kind of human activity without restrictions is recommended.

**Comments:** The anatomic and functional normality, after correction of the pulmonary valve stenosis, may only be obtained through proper surgical conduct, under direct vision, in valve anatomy without dystrophies and well constituted valves, as well as a normal sized pulmonary annulus. This surgical idea maintains that percutaneous commissurotomy with rupture of the valve, and not of the commissure, generally results in a less favorable evolution, especially if it is related to varied degrees of pulmonary valve insufficiency. However, according to reports found in the literature, more deeply marked degrees of pulmonary valve insufficiency occur in 20 to 30% of cases and require further surgical procedures with placement of a biological valve, regardless of the previously employed technique, surgical or percutaneous. Thus the percutaneous procedure became routine in clinical practice.^1,2^ Most patients who undergo percutaneous treatment evolve favorably, since pulmonary valve insufficiency does not occur in 12%, is mild in 64%, moderate in 18%, and severe in 6%.^2^ The results, in general, are comparable between the two procedures, surgical or percutaneous. Thus we can state than the best evolution is related to a more adequate anatomy of the pulmonary valve, especially when a commissurotomy is flawlessly performed, surgically or percutaneously.
